# O6-1 Exploring the association between green space attributes and physical activity to inform urban planning and policy

**DOI:** 10.1093/eurpub/ckac094.041

**Published:** 2022-08-29

**Authors:** Ina Šuklje Erjavec, Jana Kozamernik, Vita Žlender

**Affiliations:** Urban Planning Institute of the Republic of Slovenia, Ljubljana, Slovenia

**Keywords:** active lifestyle, quality of life, green space, urban planning guidelines, cross-sectoral approach

## Abstract

Publicly accessible green open spaces such as parks, playgrounds and urban forests are key environments within urban tissue for promotion of health-enhancing physical activities (HEPA). The association between green open space and physical activity has been a subject of numerous studies, most of them focusing on factors affecting the use of green space for HEPA. However, there are inconsistencies among studies about associations between spatial characteristics of a public open space (e.g. size, distance, safety, quality) and HEPA. This presents a struggle for urban designers and policy makers to develop and implement evidence-based guidelines for designing green open spaces to promote HEPA. Slovenian planning and management plans are no exception to this. In order to effectively overcome this shortfall, we developed an approach and methodology combining current knowledge, planning evaluation and an empirical study.

Accordingly, we conducted a comprehensive analysis of current national legislation and documents related to public health, physical activity promotion and spatial planning. We also carried out workshops, focus groups, and discussions with relevant public health experts, decision makers and planners. To assess the practical relevance of the factors identified by experts and research literature, we tested the proposed approach and guidelines on a pilot study in the municipality of Kocevje, which is the largest Slovenian municipality, characterized by one town (Kocevje) and a number of small villages. Pilot study encompassed analysis of the municipality documents and spatial plans, spatial analyses, a survey among 176 residents of the municipality, and interviews with relevant representatives of local authority.

The analysis of the planning plans showed that there is not one single official document which would stress the importance of green open space for HEPA, either on strategic or implemental level. Furthermore, the pilot study proved great differences between the town and surrounding villages, the town being insufficient in supply of green spaces suitable for walking or cycling, whilst villages were lacking open spaces as a setting where people could engage in physical activity.

Based on the results, we underline the importance of the topic integration into planning documents and propose guidelines to achieve this.

